# A co-design process developing heuristics for practitioners providing end of life care for people with dementia

**DOI:** 10.1186/s12904-016-0146-z

**Published:** 2016-08-02

**Authors:** Nathan Davies, Rammya Mathew, Jane Wilcock, Jill Manthorpe, Elizabeth L. Sampson, Kethakie Lamahewa, Steve Iliffe

**Affiliations:** 1 Research Department of Primary Care & Population Health, University College London, Royal Free Campus, Rowland Hill Street, London, NW3 2PF UK; 2 Social Care Workforce Research Unit, King’s College London, London, WC2B 6NR UK; 3 Division of Psychiatry, Marie Curie Palliative Care Research Department, University College London, 6th Floor, Wing B, Maple House, 149 Tottenham Court Road, London, W1T 7NF UK; 4 Barnet Enfield and Haringey Mental Health Trust Liaison Team, North Middlesex University Hospital, Sterling Way, London, N18 1QX UK

**Keywords:** Dementia, End-of-life care, Palliative care, Co-design, Qualitative research

## Abstract

**Background:**

The end of life for someone with dementia can present many challenges for practitioners; such as, providing care if there are swallowing difficulties. This study aimed to develop a toolkit of heuristics (rules-of-thumb) to aid practitioners making end-of-life care decisions for people with dementia.

**Methods:**

An iterative co-design approach was adopted using a literature review and qualitative methods, including; 1) qualitative interviews and focus groups with family carers and 2) focus groups with health and care professionals. Family carers were recruited from a national charity, purposively sampling those with experience of end-of-life care for a person with dementia. Health and care professionals were purposively sampled to include a broad range of expertise including; general practitioners, palliative care specialists, and geriatricians. A co-design group was established consisting of health and social care experts and family carers, to synthesise the findings from the qualitative work and produce a toolkit of heuristics to be tested in practice.

**Results:**

Four broad areas were identified as requiring complex decisions at the end of life; 1) eating/swallowing difficulties, 2) agitation/restlessness, 3) ending life-sustaining treatment, and 4) providing “routine care” at the end of life. Each topic became a heuristic consisting of rules arranged into flowcharts. Eating/swallowing difficulties have three rules; ensuring eating/swallowing difficulties do not come as a surprise, considering if the situation is an emergency, and considering ‘comfort feeding’ only versus time-trialled artificial feeding. Agitation/restlessness encourages a holistic approach, considering the environment, physical causes, and the carer’s wellbeing. Ending life-sustaining treatment supports practitioners through a process of considering the benefits of treatment versus quality-of-life and comfort. Finally, a heuristic on providing routine care such as bathing, prompts practitioners to consider adapting the delivery of care, in order to promote comfort and dignity at the end of life.

**Conclusions:**

The heuristics are easy to use and remember, offering a novel approach to decision making for dementia end-of-life care. They have the potential to be used alongside existing end-of-life care recommendations, adding more readily available practical assistance. This is the first study to synthesise experience and existing evidence into easy-to-use heuristics for dementia end-of-life care.

**Electronic supplementary material:**

The online version of this article (doi:10.1186/s12904-016-0146-z) contains supplementary material, which is available to authorized users.

## Background

Dementia is one of the biggest health concerns facing older people and health and social care systems across the world and has become an international public health priority [1]. Despite emerging epidemiological evidence that the incidence of dementia is declining, [2] the prevalence of dementia is still rising across the world because of the ageing population [3–5].

Typically, as people with dementia approach the end of life they develop symptoms which can be distressing and create dilemmas for practitioners and family members [6]. These may include difficulties with swallowing and therefore problems with eating, drinking and taking oral medication, agitation, a weakened immune system leading to susceptibility to infections, skin breakdown, and shortness of breath [7]. The deterioration experienced at the end of life may be similar to that experienced in other terminal diseases such as some cancers. However, people with dementia not only experience these symptoms for a longer period of time with uncertain prognosis, [8, 9] but may not have the cognitive capacity to verbally express their symptoms or make decisions regarding their own care. This, in turn, makes the processes of recognition, treatment and decision-making more challenging.

Clinical decision-making in England is informed by professional beliefs about best practice, available scientific evidence, and clinical guidelines such as those produced by the National Institute for Health and Care Excellence (NICE), [10–13] as well as by specialist organisations such as the Alzheimer’s Society and the National Council for Palliative Care (NCPC) [14].

Until recently NICE’s national guidelines on palliative and end of life care focussed on cancer, [10] with quality standards and minimal guidance for end of life care for people with dementia [11, 12, 15, 16]. New guidelines published in December 2015 have a broader focus on ‘dying adults’, in the last few days of life [13]. Many researchers and practitioners acknowledge that palliative care for dementia may span a much longer period of time, [17] starting earlier in the course of the condition, with end of life care not restricted to just the last few days or even the last 12 months, but potentially a period of years [18–21].

The lack of guidance specific to end of life care for people with dementia has been made more problematic by the withdrawal of the Liverpool Care Pathway. The removal has left practitioners without a framework for providing end of life care in the terminal phase, having demonstrated in previous studies its use as a framework for providing end of life care for people with dementia, [22] and elderly people at the end of life in non-cancer settings [23]. This has reportedly had a negative impact on the confidence of many practitioners, even those who are experienced in providing end of life care [24]. However, at an international level the European Association for Palliative Care (EAPC) has defined optimal palliative care for people with dementia using a consensus-based approach [17]. This covers 11 domains with 57 recommendations, providing a framework for guidance encompassing clinical practice, policy and research.

Many previous clinical guidelines regarding end of life care for people with dementia were limited by a lack of evidence [25]. There is a paucity of randomised controlled trials (RCT) to support recommendations, many of which had small sample sizes, lacked strong underpinning theoretical development limiting the robustness of the findings [26]. In this context, heuristics (‘rules of thumb’) have been proposed as an alternative to the use of guidelines to facilitate decision-making by health and care professionals [27]. Heuristics are schematic patterns that can be applied in complex situations and function as prompts to initiate thinking and action; they offer a clinically familiar approach, are brief, easy to remember and lead to action. Heuristics are simple decision aids that can be more accurate than other complex, ‘information-greedy’ classification and prediction tools [28]. They are transparent, speedy and not reliant on technology. Because they use less information and are fast, they are efficient, making them particularly useful for conditions of uncertainty in which decisions have to be made without delay [28].

It is assumed that when practitioners make decisions, they employ a systematic approach which involves weighing up all the options, and the pros and cons which accompany each of these options. However, non-systematic processes are common, [29] in particular when making decisions about health [30]. Those making decisions in clinical practice often use heuristic strategies [31]. The heuristics that general practitioners use in making clinical decisions can shape performance more powerfully than any form of formal training. [32] An example of this is ‘Sutton’s Law’, which advocates considering a common diagnosis to explain symptoms before considering an uncommon one [33].

**Fig. 1:**
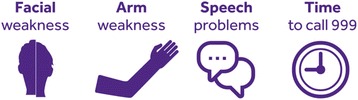
Stroke heuristic FAST (Taken with permission from the Stroke Association website www.stroke.org.uk)

An example of a successfully implemented health-related heuristic in the public domain is ‘FAST’, designed to cue people about recognising stroke symptoms (see Fig. 1).[34] This is a tallying heuristic, which weights all cues equally and counts cues favouring one alternative in comparison to others [35]. It can be understood as a rule of thumb:

Facial weakness + Arm weakness + Slurred speech = time to call for an ambulance

The study described here aimed to develop a toolkit of heuristics to help practitioners with decision-making at end of life for people with dementia. This paper will describe the development process for the heuristics, which are being evaluated for utility and impact in different settings [20].

## Methods

### Design

**Fig. 2:**
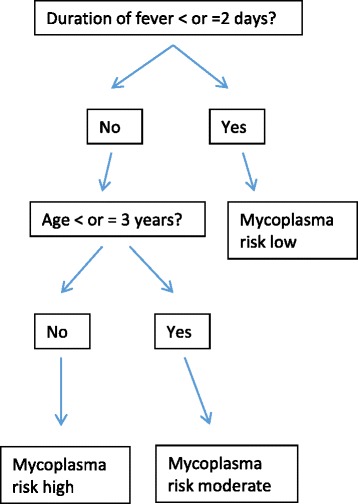
Decision tree for treatment with macrolide antibiotics of community acquired pneumonia attributable to Mycoplasma pneumoniae in children

Using information from three sources and applying an iterative co-design approach, triangulation of data was carried out using a literature review, qualitative interviews and focus groups with family carers (‘experts by experience’), and focus groups with health and care professionals. Interviews were only conducted with family carers as some family carers expressed that they did not wish to take part in focus groups. Co-design is a technique adopted from product development [36] which has tangible benefits in developing or redesigning health services [37–40]. The co-design task was to develop heuristics with only few attributes that operate under three rules: 1) search through cues in a pre-determined order; 2) stop searching as soon as a cue leads to an exit; and 3) classify the object of concern accordingly [35]. An example is shown in Fig. 2, showing a decision tree for treatment of community acquired pneumonia attributable to Mycoplasma pneumoniae in children with macrolide antibiotics [28]. The branches of the tree show the pre-determined order of questioning, the object of concern is Mycoplasma pneumonia and three levels of risk represent the exits from the decision tree.

### Participants and recruitment

#### Family carers

Former and current family carers of people with dementia were recruited to ensure a range of experiences and opinions based on previous and current experiences, from the Alzheimer’s Society Research Network, using a purposive sampling approach, selecting those with experience of providing end of life care for a person with dementia at home or care in hospital. Carers with experience at home or hospital was specified as the heuristics were being developed for these settings.

#### Health and care professionals

Due to a multitude of potential complications and symptoms at the end of life for someone with dementia, we purposively sampled a broad range of health and care professionals with varying experiences and expertise working with people with dementia at the end of life, including; general practitioners, palliative care nurses and physicians, geriatricians, speech and language therapists, hospital nurses, healthcare assistants, community nurses, and pharmacists. Professionals were recruited through the Dementias and Neurodegenerative Diseases Research Network (DeNDRoN) co-ordinating centre and the Comprehensive Local Research Network (CLRN). The research team also utilised its contacts within this field to identify interested health and care professionals, using a snowballing technique [41]. Snowballing is a method which uses contacts of existing participants to identify further additional participants who may be more difficult to reach through traditional methods of recruitment, such as some of the participants in this study.

#### Inclusion and exclusion criteria

- Family carers 18 years or older
- Family carers considered a primary carer for a person with dementia
- Family carers with experience of caring for someone at home and/or hospital
- Carers suffered bereavement within the last 3 months were not eligible
- Carers unable to speak English were not eligible
- Practitioners in a caring role either health or social care, for someone with dementia

### Procedure

**Fig. 3:**
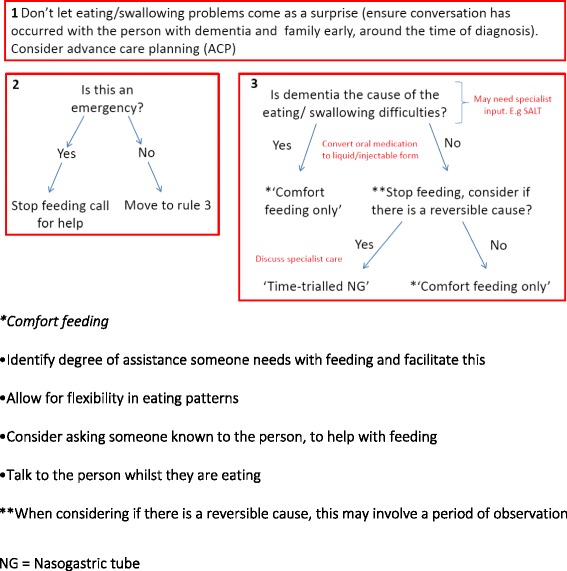
Eating/swallowing difficulties heuristic

An overview of the procedure is shown in Fig. 3. A rapid appraisal literature review was conducted to identify key areas of decision-making concerning people with dementia at the end of life [42]. The findings of the review, together with findings from a preliminary study which interviewed family carers about their ideas of quality end of life care for people with dementia, [18] were used to develop a topic guide (see Additional file 1).

The topic guide was used to facilitate focus groups and interviews with both former and current family carers of people with dementia, as well as groups of health and care professionals. The topic guide for the interviews and focus groups explored six broad areas of difficult decision making; 1) difficulties with swallowing and problems with eating, 2) agitation/comfort, 3) ending life sustaining medical treatment, 4) personhood, 5) stopping routine care (i.e. bathing and turning of a bed bound person), and 6) communication between professionals. Additionally, carers were asked to suggest further difficult decisions which needed to be made at the end of life.

The focus groups used a ‘think aloud’ strategy; participants were presented with each topic and the group were asked to vocalise their thoughts about how decisions regarding this topic are currently and should be made [43]. Traditional semi-structured interviews give people the time and chance to process and rationalise thoughts and decisions, and therefore do not necessarily give an indication of what they really think or provide any understanding of how they arrived at their decision [44]. In order to make sense of what people think and why they make the decisions they do, many have argued that we need to pay attention to verbalizations and thought processes as these highlight how information is stored in working memory [43, 45, 46]. Working memory contains the thoughts in the present moment as well as the memories which are being retrieved from long-term memory [47]. The think aloud strategy allows us to hear and access thoughts in working memory and hence get a better understanding of how individuals make decisions. The ‘think-aloud’ method was developed for the usability testing of technical products but has been used to understand the complex process of decision making in healthcare settings [45].

**Fig. 4:**
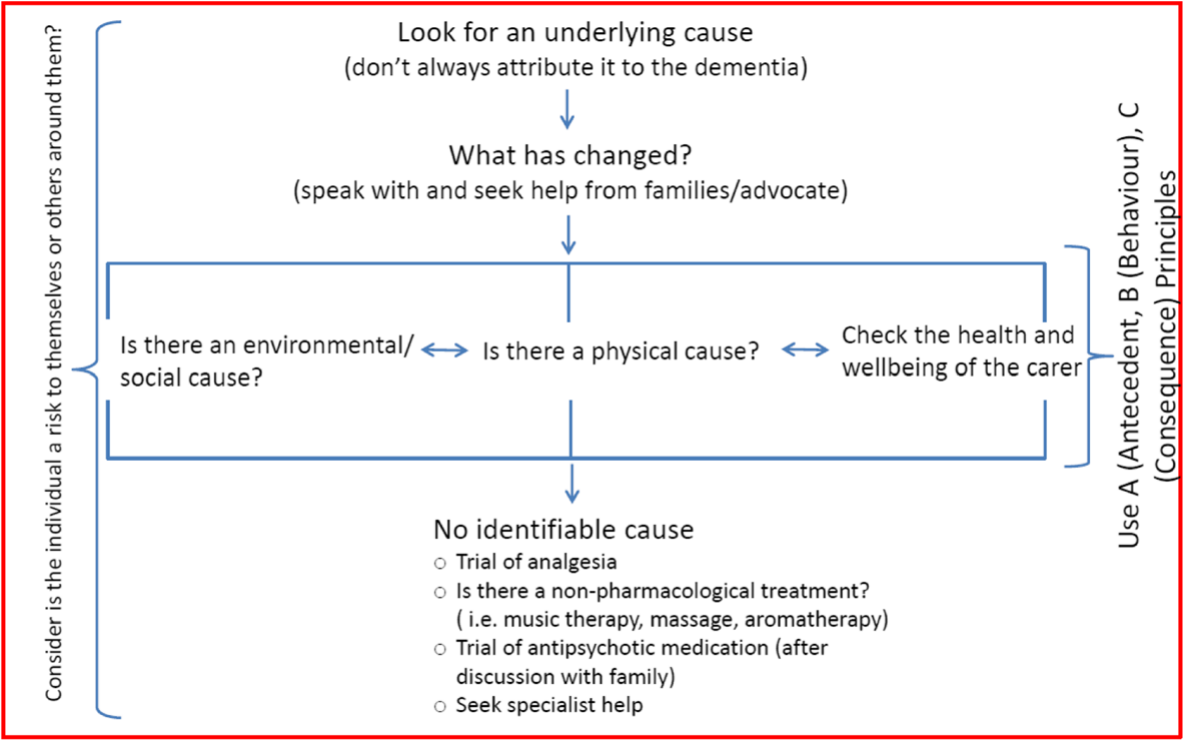
Agitation/restlessness heuristic

**Fig. 5:**
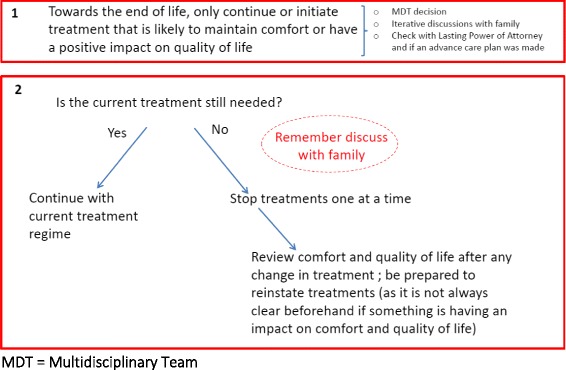
Ending life sustaining treatment heuristic

**Fig. 6:**
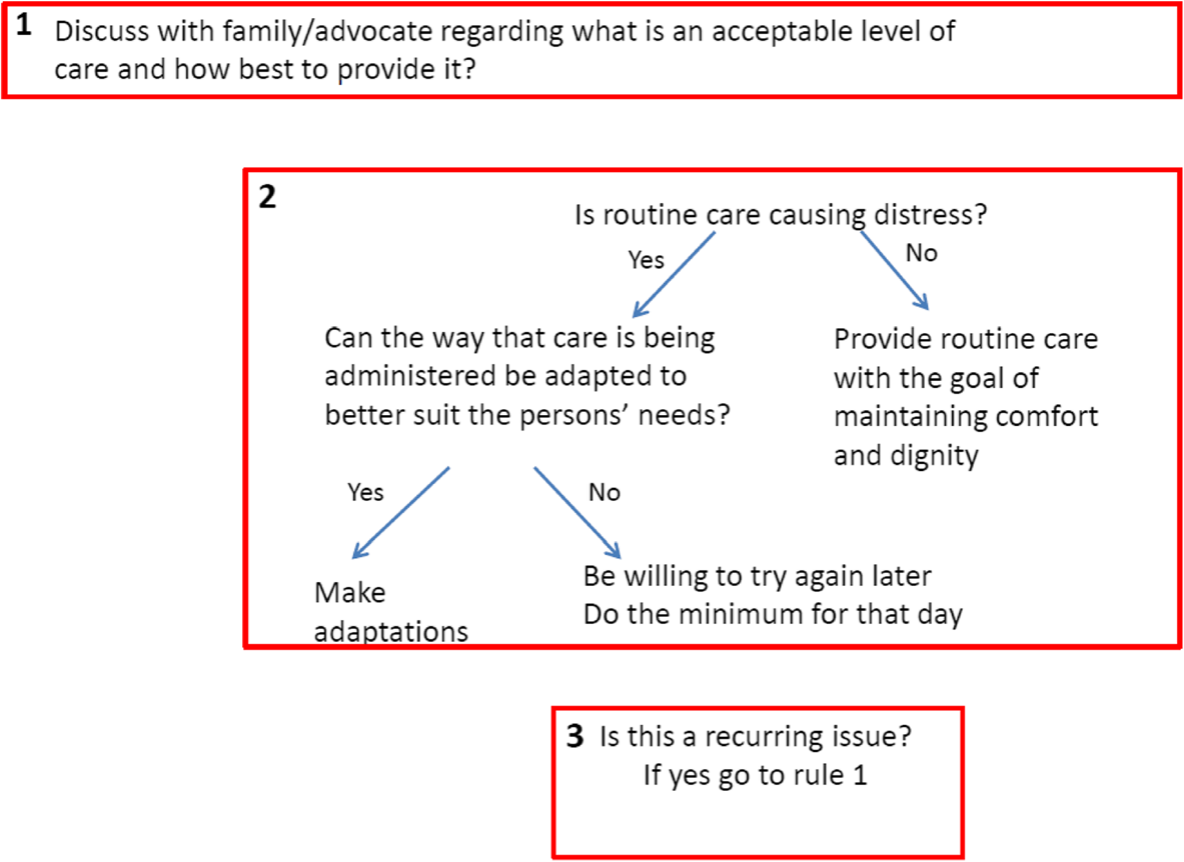
Providing routine care at the end of life heuristic

**Fig. 7:**
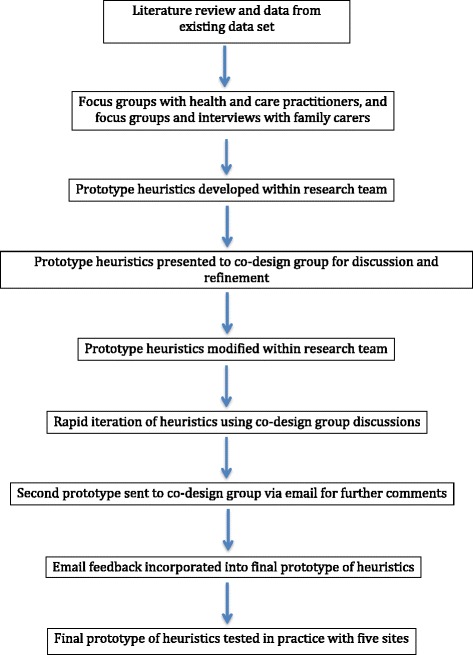
Overview of co-design process

Findings from the interviews, focus groups and the literature review informed the development of a toolkit of heuristics. Transcripts were read by four members of the research team (ND, SI, RM, JW) and key messages and decisions/decision processes were recorded by each researcher. The research team met to discuss all key messages from the data and how these may reflect heuristics. From this initial meeting a series of heuristics consisting of statements were generated. Two members of the team (ND and RM) constructed heuristics from these statements as flowcharts as shown in Figs. 4, 5, 6 and 7. The research team met again to discuss the developed heuristics in order to refine them, using the interviews and review of the literature as a basis for this, together with their clinical and research experiences.

To develop the heuristics further a co-design group was established. The co-design group consisted of health and social care practitioners (palliative care consultant, two GPs, Admiral (specialist dementia) nurse, social care professional, two geriatricians, and a community nurse), four family carers and members of the research team with backgrounds in psychology, social care, anthropology, and general practice. The co-design groups’ expert knowledge and interpretation of the available evidence was used to further develop the heuristics prior to their testing in real settings.

The co-design group met following analysis of findings from the focus groups and interviews to discuss the development of the heuristics, using a nominal group process. Nominal groups are structured meetings with an aim to facilitate group thinking and decision making about a given problem within an expert group. A nominal group process was chosen as it has been shown to have a useful role in analysing health care problems [48], and can help bridge the gap between researchers and practitioners [49]. Such groups keep experts’ time commitment to a minimum and collect a variety of ideas whilst allowing for interaction and discussion of these ideas.

The group were presented with an overview of the evidence from both the literature review and qualitative data, using a PowerPoint presentation. Each member of the group was given an opportunity to ask questions of clarification and detail from the team. Following the presentation each member of the team was provided with the provisional heuristics, as both written statements and flowcharts. Members of the group were initially asked to read through these independently and then bring thoughts back to the group about the presentation of these heuristics. Specifically members of the group were asked their preferred format: visual image or written statements. Following this, each topic was discussed in detail. The research team presented each topic individually and asked specific questions of the group to promote discussion and facilitate problem solving. Examples of questions and the answers to these questions, with implications for changes to the heuristics, are discussed below.

Detailed notes were taken of the nominal group process with the co-design group of comments and discussions which were made and the conclusions drawn from these discussions. These notes were then used by the research team to inform a rapid iteration following the meeting. The nominal group outputs were sent by email to all members of the co-design group for further comment and feedback on the heuristics. A near-final prototype of each heuristic was revised by the research team and sent to the members of the co-design group, prior to testing in real settings.

## Results

This section presents the final prototype of heuristics devised with the co-design group and presented to five sites to use in their clinical practice with people with dementia who are recognised to be at the end of life. These sites included; one general practice, two community palliative care teams, one community nursing team, and one hospital care of the elderly ward. Findings from the implementation and evaluation of the heuristics will be reported separately.

### Heuristics

Key topics regarding difficult decision making were considered to be: eating and swallowing difficulties, agitation/restlessness, ending life sustaining treatment, and providing routine care at the end of life. Other topics, including person-centred care and communication between professionals, were deemed by the co-design group to be less appropriately addressed by heuristics and also more ‘generic’ to good dementia care rather than specific to end of life care.

#### Eating/swallowing difficulties

The heuristic for eating and swallowing difficulties was devised using three rules which are interconnected, each represented by a red box in Fig. 4. Rule one represents the need to have early conversations about the possibility of eating difficulties towards the end of life, in order to ensure that this problem does not come as a surprise to families and others involved in decision-making.

The second rule applies when the person with dementia is no longer able to eat or swallow and asks the professional to consider if this is an emergency situation (e.g. is the individual choking?). This highlights that it is appropriate in this situation to call the emergency services (ambulance/paramedic service) and not fear that this will be seen as an unnecessary hospital admission.

The final rule applies if the swallowing difficulty is not considered to be an emergency, and asks the professional/s to assess if the difficulties are due to a progression of the dementia or an alternative cause. This may require input from a Speech and Language Therapist (SALT) or other specialist. If reduced eating or swallowing difficulties are deemed to be a consequence of dementia, the rule is to advocate ‘comfort feeding only’. Comfort feeding only, which is used in place of ‘at-risk feeding’, simply refers to the process of eating for pleasure, providing small amounts of food; the risk of aspiration should be balanced with the potential for comfort and pleasure that eating may provide [50]. At this stage all oral medication should be converted to liquid form, if this has not already been done.

In any situation in which swallowing and eating difficulties are not thought to be due to the dementia, there needs to be an assessment of whether the cause is potentially reversible. For example, an infection or mouth soreness could be treated with antibiotics or simple mouth care. At this time, discussion with a specialist (for example, a geriatrician) may be needed to assess the appropriateness of nasogastric tube feeding (NG) while the reversible cause is being treated. Finally, a non-reversible cause would direct the decision to comfort feeding only.

During the development of this heuristic the co-design group was specifically asked to provide expertise about when to have discussions about the difficulties in swallowing and eating. The group decided that this should be part of the advance care planning process and done as early as possible. “Comfort feeding only” was also raised in the group which was asked if this terminology was appropriate [50]. The group felt that this was a difficult concept to get right however, they felt this terminology was better than the nearest equivalent term of ‘at-risk feeding’. The most striking change after group consultation and discussion was the separation of one rule into three rules to form the heuristic.

#### Agitation/restlessness

Unlike the first heuristic, agitation and restlessness are in one red box to symbolise one rule in its entirety. The broad message from this heuristic is that agitation or restlessness should not always be attributed to dementia (see Fig. 5).

This heuristic encourages professionals to engage with families and understand what has changed in the individual’s life and/or care. The heuristic focuses on three areas which should be considered simultaneously rather than in a hierarchical fashion; environmental and social changes, physical causes, and the general health and wellbeing of the family carer. Further guidance is provided on what may be the cause under each of these categories on a separate page, for example physical causes may include pain, hunger/thirst or constipation.

Prior to discussions with the co-design group, the flow of this heuristic was very complicated. The group simplified the design of the heuristic and emphasised the importance of considering all three areas equally. Finally, the group agreed it was important to highlight that there may not be an identifiable cause and this was acceptable and should not be seen as a failure by the medical, nursing, or care team.

### Ending life sustaining treatment

Heuristic three consists of two rules which may help with decisions for ending life sustaining treatment and initiating new forms of treatment (see Fig. 6). Life sustaining treatment was considered to be any treatment that was not solely about symptoms and that had long-term benefits, such as statins or antihypertensives. The first rule encourages an iterative process of discussions with family members and a multi-disciplinary team approach, whereby treatment is only continued or initiated if it maintains comfort or quality of life.

The second rule focuses on how to proceed with rationalising medication and determining what treatments are still needed at the end of life. Decision-makers are advised to only continue or initiate treatments that are likely to maintain comfort or have an impact on quality of life. The rule advocates removing treatments one by one, in order to avoid ambiguity about any possible adverse effects following their withdrawal. Subsequently, following the removal of any treatment, quality of life and comfort should be reassessed and the professional should be prepared to reinstate treatments if appropriate.

Discussions with the co-design group did not lead to significant changes in this heuristic. There was a group emphasis that this heuristic should not include antibiotic treatment. It was felt that whether or not to initiate antibiotics was an acute treatment decision that required its own decision-making framework. Specifically, it was not felt to be comparable with decisions about whether or not to continue statins and antihypertensives.

### Providing routine care at the end of life

Providing “routine care” encompassed several aspects including personal care (i.e. bathing and grooming) as well as turning an individual who is bed bound as a preventative measure for pressure ulcers (see Fig. 7). However, routine care does not include turning or moving a patient if they already have a pressure ulcer and need to be turned to relieve discomfort. This rule, unlike many of the others, applies to the final few days and hours of life, when providing routine care may be uncomfortable and unnecessary for the individual.

The heuristics consists of three rules which prompt professionals to discuss with the family or advocate about what an acceptable level of care may be. For example, there should be discussions with relatives about whether the patient would like their bed sheets to be changed if they were not wet or soiled (wet and soiled sheets/clothing would always still be changed).

The second rule which will be informed by these initial discussions encourages the professional to consider if routine care is causing distress and if so make amendments or provide an agreed minimal level of care without causing distress. Finally, rule three highlights that if distress due to routine care is recurring, then further discussions with the family about an acceptable level of care are recommended.

The co-design group felt it was important to emphasise at the start of this heuristic the need to discuss with the family/advocate about what they feel is an acceptable level of care. The original heuristic presented to the co-design group placed discussions with the family at the end after problems around providing routine care were identified. The co-design group also felt this heuristic was better suited to being deconstructed into three separate rules.

## Discussion

### Key findings

We used an iterative co-design process to develop a toolkit of four heuristics on important and complicated problems which practitioners working in end of life care for people with dementia find challenging. We combined information from the existing literature, together with the perspectives of family carers and practitioners from a range of disciplines, backgrounds and experience in a technology development method to synthesise evidence based heuristics.

The four heuristics developed in this study have been developed with the aim of being used by health and care practitioners, including doctors, nurses and care practitioners in different settings. They provide a flowchart of rules (accompanied by additional written material) to help practitioners make informed decisions with more confidence.

We would suggest that the heuristics have the potential to be used alongside the international EAPC recommendations of optimal palliative care for people with dementia [17]. The EAPC recommendations provide a comprehensive overview of care and what palliative care for someone with dementia should look like, whilst heuristics provide more practical assistance to guide practitioners through making difficult and complex decisions.

### Areas for consideration

The development process raised questions about which audiences and settings are the most appropriate for the use of heuristics. As others argue, some heuristics will be more appropriate for nursing and social care and others for medical care [51]. The same applies to the setting in which these heuristics may be appropriate, for example providing routine care would be applicable across all relevant settings, community, care home, hospital or hospice. However, aspects of the eating and swallowing difficulties heuristic may not be applicable to community teams, who are unlikely to insert nasogastric (NG) tubes in the home, although they may support community dwelling people with them in situ, although this may also vary across different countries. Research from the USA suggests there is no evidence that artificial feeding increases quality of life, [52] and a general consensus from International EAPC recommendations that permanent artificial feeding should be avoided [17].

The discussions about heuristics in this development process have also highlighted the need for caution in their use. Although heuristics have been described as a form of ‘fast and frugal’ decision making which frequently leads to the right answer [53], not all end of life decisions can be made quickly. Some decisions will require careful consideration, good communication within teams and between professionals and families, checking advance care plans and a multi-disciplinary team approach - for example, stopping life sustaining treatment. However, heuristics can in some instances help create a logical framework for making fast decisions which lead to action, such as the provision of routine care.

Some of the heuristics such as agitation/restlessness and eating/swallowing difficulties assume that specialist help such as a specialist palliative care team, geriatrician, or old age psychiatrist is available, however not all teams particularly in the community where many people with dementia reside will have access to such help. For example, the heuristic regarding agitation/restlessness suggests seeking specialist help from an old age psychiatrist if there is no identifiable cause of agitation/restlessness. However, again this may vary across countries and health care systems.

Despite the attraction of heuristics being a simple, efficient, fast method particularly useful for conditions of uncertainty, [28] they are mental shortcuts and therefore there are limitations in their use; in particular they can lead to faulty conclusions and cognitive biases [54]. One particular example of cognitive bias is the concept of ‘representativeness’, which refers to errors made because thinking is overly influenced by what is typically true. For instance, when someone with advanced dementia begins to refuse food, this may be due to the disease process itself but it can also be due to an entirely separate physical or even psychological cause, which may be overlooked and reversible, for example oral thrush. The heuristics presented in this paper are transparent and therefore minimise error in judgement and resulting bias, but they do not eliminate error. Throughout the heuristics process, the importance of discussions with the family, nominated decision maker and person with dementia (if able) is highlighted. Discussions should happen as early as possible as part of an approach to advance care planning. However, this may not always be possible as some people with dementia and their families do not wish to discuss their own mortality and end of life care plans [18, 19, 55].

### Strengths and limitations

A strength of this study is the direct focus it has placed on the views of family carers and those who make difficult decisions about end-of-life care for people with dementia. The co-design group contained a mix of health and care practitioners and former family carers which allowed for detailed discussions about the heuristics and their content, increasing the rigour in the development process which has been highlighted as often lacking in previous research within this field [26].

This study is limited by the number of rounds (two) of iteration that the heuristics received; more rounds of discussion with the co-design group may have enhanced the usability and design of the heuristics. However, further development work is planned in subsequent phases of the study.

### Future research

The developed heuristics are being used by five different teams including one general practice, one community nursing team, an older adult hospital ward, and two community palliative care teams [20]. The heuristics will be used in practice by these five teams and evaluated after three and 6 months of use. Some alterations and refinements of the heuristics will likely be made with the co-design group using a similar procedure to that described in this paper at the 3 month stage. Finally, at the close of the study, the co-design group will help produce a finalised version of the heuristics.

## Conclusions

Heuristics are a novel approach to decision making for dementia end-of-life care, despite being already used in many other aspects of health care decision-making. It is hoped that the heuristics described in this paper will provide practitioners with a practical toolkit to make difficult decisions about complex situations.

## Abbreviations

CLRN, Clinical Local Research Network; DeNDRoN, Dementias and Neurodegenerative Diseases Research Network; EAPC, European Association for Palliative Care; GP, General Practitioner; MDT, Multidisciplinary Team ; NG, Nasogastric Tube; RCT, Randomised Controlled Trial; SALT, Speech and Language Therapist ; USA, United States of America.

## Additional file

Additional file 1: Topic guide. Copy of topic guide used in focus groups and interviews. (DOCX 19 kb)
