# Erratum to: A co-design process developing heuristics for practitioners providing end of life care for people with dementia

**DOI:** 10.1186/s12904-016-0148-x

**Published:** 2016-08-18

**Authors:** Nathan Davies, Rammya Mathew, Jane Wilcock, Jill Manthorpe, Elizabeth L. Sampson, Kethakie Lamahewa, Steve Iliffe

**Affiliations:** 1 Research Department of Primary Care & Population Health, University College London, Royal Free Campus, Rowland Hill Street, London, NW3 2PF UK; 2 Social Care Workforce Research Unit, King’s College London, London, WC2B 6NR UK; 3 Division of Psychiatry, Marie Curie Palliative Care Research Department, University College London, 6th Floor, Wing B, Maple House, 149 Tottenham Court Road, London, W1T 7NF UK; 4 Barnet Enfield and Haringey Mental Health Trust Liaison Team, North Middlesex University Hospital, Sterling Way, London, N18 1QX UK

## Erratum

Upon publication of the original article [1], the authors noticed that the numbers of figures 3–7 had been assigned to each figure and its label incorrectly. In the original article:

“Figure 7” should have read “Figure 3” (Overview of co-design process)

“Figure 3” should have read “Figure 4” (Eating/swallowing difficulties heuristic)

“Figure 4” should have read “Figure 5” (Agitation/restlessness heuristic)

“Figure 5” should have read “Figure 6” (Ending life sustaining treatment heuristic)

“Figure 6” should have read “Figure 7” (Providing routine care at the end of life heuristic)

These figure numbers have now been updated in the original paper.

We, the publishers, apologise for any inconvenience caused by these errors and would like to thank Dr Nathan Davies from University College London for bringing this to our attention.
