# Supplementary material for: A co-design process developing heuristics for practitioners providing end of life care for people with dementia
Source: BMC Palliat Care. 2016 Aug 2;15:68. doi: 10.1186/s12904-016-0146-z (PMC4969644; doi:10.1186/s12904-016-0146-z)
Supplement: Additional file 1: — Topic guide. Copy of topic guide used in focus groups and interviews. (DOCX 19 kb) [file 12904_2016_146_MOESM1_ESM.docx]

**Supplementary file 1: Topic guide**

***Stage 1.****Introduction (5min): An introduction from the facilitator, explaining the purpose of the study and the focus groups, with some ‘ground rules’ for the discussions, at which point the recorder will be switched on. The facilitator will invite participants to introduce themselves.*

**Introduce observer(s)**

**Aims for the group discussion**

You have all been sent a copy of the participant information sheet but we’re just going to give you a brief overview of the study.

This study is developing and testing some heuristics (rules of thumb) for people with dementia at end of life.

Heuristics are basically rules of thumb, decision aids or even simple statements which enable quick decision making about complex issues and situations.

An example is FAST which is used to identify the signs of a stroke with stroke patients; **F**acial drooping, **A**rm weakness, **S**peech difficulties, **T**elephone 999. [PPT1-PLEASE SHOW ON SCREEN]

We have asked you here today because you have experience with end of life care of people with dementia. We want to know what you think are the most important decisions that need to be made at the end of life, and how you would approach or think these decisions should be made.

There are no right or wrong answers - we are interested to hear your thoughts and to learn from your experiences.

We have some topics that we would like to go through with you based on our previous research with family carers and a review of the literature. At the end we would like to hear anything that you think is important that we have not discussed or maybe what we think may be important but actually is not.

**Remind participants about confidentiality and audio recording**

We’d like to record the discussion if that’s okay with you? Anything you say here will be anonymised and won’t be linked with your name. All the information will be stored securely and only the researchers working on this study will have access to them. All the recordings will be destroyed at the end of the study.

*Does anybody have any questions?*

**Check all have read the information sheet and signed the consent form**

A bit about how today is going to run – The entire group should last no more than 60-90min, if anyone wants to stop or leave you can do so at any time, etc. Also please help yourself to lunch and drinks throughout, we want this to be a very informal group discussion. You will be able to speak to one of us privately at the end if there is something you would rather talk about one-on-one.

**Start Group Discussion**

We would like to go round the group and if everyone can introduce themselves with the name they would like to be used in the discussion if you do not wish to use your own name. It would also be useful if you could say a little bit about your experience of caring for someone with dementia.

***Stage 2.*** *Opening the topic (5min): The facilitator will introduce quality of care ideas and up to 10 cases or heuristics developed from the results of previous 47 in-depth interviews with family carers and literature review.*

***Stage 3*** *Discussion (think-aloud) (up to 50min): The topic will be displayed on a screen individually for up to ten minutes each (see below) and participants will be asked to discuss, critique and think of ways to change or improve on the heuristics, whilst verbalising their thought processes. The facilitator/observer will record key ideas on the flipchart.*

1. ***Difficulty in swallowing and problems with eating*** [PPT2]

At the end of life swallowing can become more difficult and there is sometimes a desire to try and help for example, with the use of tube feeding.

- Would someone like to start by sharing their experiences?
- How did you approach that?
- What were the decisions that you needed to make here?
- What are the right decisions?
- Can we write a rule to capture the right decision?
- Would this decision be different across different settings?

1. ***Agitation/comfort*** [PPT3]

Agitation and the management of agitation with people with dementia can sometimes be poor. We want to explore how agitation should and could be identified and subsequently managed.

- What do you do when someone becomes agitated?
- Does anyone have a good example of how agitation was managed in their experience?
- What decisions need to be made here?
- What are the right decisions?
- Can we write a rule to capture the right decision?
- Would this decision be different across different settings?

1. ***Medical treatment and ending life sustaining medical treatment*** [PPT4]

We would like to explore medical treatment that is maintaining the life of a person with dementia and when is it appropriate to cease such medical treatment. Examples of this kind of treatment may include treatment for co-morbidities such as diabetes, treatment for infections such as pneumonia, stroke, pace makers.

- When is it right to stop life sustaining medical treatment?
- What decisions need to be made here?
- What are the right decisions?
- Can we write a rule to capture the right decision?
- Would this decision be different across different settings?

1. ***Personhood*** [PPT5]

Personhood is a standing or status that is bestowed upon one human being, by others, in the context of relationship and social wellbeing. To be simply viewed as ‘someone with dementia’ or just a label would imply a lack of personhood and a reduction in their social status or standing, and they are no longer a ‘person’. We would like to explore the maintenance of personhood of the individual with dementia at the end of life.

- Would someone like to start by sharing their experiences?
- What are the important things we need to consider to ensure personhood is maintained?
- What considerations and decisions need to be made here?
- What are the right decisions?
- Can we write a rule to capture the right decision?
- Would this decision be different across different settings?

1. ***Stopping routine care*** [PPT6]

We would like to discuss your experiences of care of a person with dementia in relation to bathing, grooming and intimate care.

- At what point should routine care be stopped?
- What decisions need to be made here?
- What are the right decisions?
- Can we write a rule to capture the right decision?
- Would this decision be different across different settings?

1. ***Communication between professionals*** [PPT7]

What are your experiences of the communication and coordination of care between different doctors, nurses and social care professionals?

- Would someone like to start by sharing their experiences?
- What decisions need to be made here?
- Are there specific decisions which need to be made in relation to transfers between settings (e.g. hospital admission or hospital discharge)?
- What are the right decisions?
- Can we write a rule to capture the right decision?
- Would this decision be different across different settings?

**Stage 4.**          *Summary and close (10min)*: The facilitator will round up the group discussion with a summary of the key topics and thoughts from the group using the flipchart. This will also be the opportunity for anything that participants would like to add that they feel has been missed. Finally thank the participants for their time and thoughts.

- **Would you like to add anything?**
- **Are there any topics we have discussed that actually you don’t think are that important?**
